# Rare fetal monster in Holstein crossbred cow

**Published:** 2013-01-20

**Authors:** A. Sharma, P. Kumar, M. Singh, N.K. Vasishta, R. Jaswal

**Affiliations:** 1 *Department of Veterinary Gynaecology and Obstetrics, College of Veterinary and Animal Sciences, CSK Himachal Pradesh Agricultural University, Palampur- 176062 (H.P.), India*; 2 *Veterinary Gynaecologist, Veterinary Polyclinics, Mandi (H.P.), India*

**Keywords:** Cow, Fetus, Holstein Friesian, Monster, Twins

## Abstract

This report describes a rare case of conjoined female twin monster (Monocephalus Thoracopagus Tetrabrachius Tetrapus Dicaudatus) in a Holstein Friesian pluriparous crossbred cow.

## Introduction

Developmental abnormalities of the ovum, embryo or fetus occur in all species of domestic animals. Monstrosity is a disturbance of the development that involves various organs and systems which can cause great distortion of the individual (Vegad, 2007).

The monstrosities are associated with either infectious disease or congenital defects (Arthur *et al.*, 2001) which may or may not interfere with birth. Abnormal duplication and/or disruption of the inner cell mass in an embryo give rise to congenital fetal abnormalities with partial duplication of body structures. Duplication of cranial portion of fetus is more common than that of caudal portion (Roberts, 2004). It is important to know various types of monsters in animals that usually cause dystocia, which cannot be easily delivered and require a cesarean section or a fetotomy most of the time (Patil *et al.*, 2004; Sharma, 2006).

The incidence of fetal monsters, though rare, was reported by Khasatiya *et al.*, 2009; Jerome *et al.*, 2010; Ravikumar *et al.*, 2012 in cows, Dhami *et al.*, 2000; Prasad *et al.*, 2006; Sharma *et al.*, 2010 in buffaloes and Pandit *et al.*, 1994 in goats. This communication reports a rare case of conjoined twin monster (Monocephalus Thoracopagus Tetrabrachius Tetrapus Dicaudatus) in a Holstein Friesian pluriparous crossbred cow.

## Case Details

A Holstein Friesian crossbred cow in its 3rd lactation was presented with a history of a prolonged second stage of labor with forceful abdominal contractions and two hind limbs protruding from the vulva. Obstetrical examination revealed also the presence of a third hind limb in vagina. Further detailed examination confirmed the presence of an abnormal fetus.

Since forced extraction was not possible, a paramedian laparohysterotomy was performed under local anesthesia (Arthur *et al.*, 2001) and a full-term dead fetal monster was extracted. The cow was treated with an injectable combination of Amoxycillin and Cloxacillin (5 mg/kg b.wt), plus an injection of Meloxicam (0.5 mg/ kg b.wt), and supportive therapy for 7 days. The sutures were removed ten days after the cesarean section. The cow showed an uneventful recovery.

The development of female conjoined twins was nearly complete. It had a single head and neck (monocephalic) with normal eyes and ears. The twins were fused in their thoracic regions (thoracopagus), and had four front legs (tetrabrachius), four hind legs (tetrapus) and two separate tails (dicaudatus). As per Roberts (2004) the condition could be classified as a monocephalus thoracophagus tetrabrachius tetrapus dicaudatus twin monster.

### Anatomical and Radiographic Findings

**Fig. 1:**
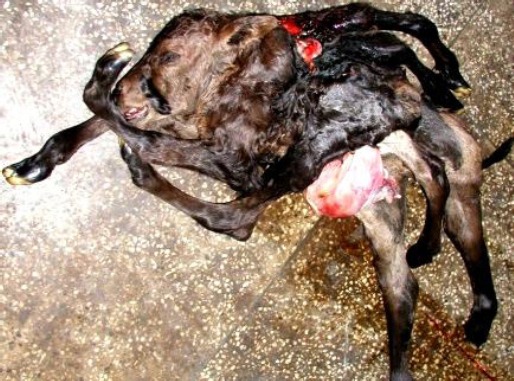
Monocephalus Thoracopagus Tetrabrachius Tetrapus Dicaudatus Monster.

The head and neck region was formed in the larger fetus only (Fig. 1). The smaller fetus was attached to the larger one at the cranial end of its abdominal cavity behind the left forelimb. Both fetuses had two fully formed forelimbs and two hind limbs each. Both fetuses had thoracic cavities but only the larger fetus had a heart and fetal lungs in its cavity. The wall of the heart was hypertrophied and the lumen of its four chambers was constricted.

The underdeveloped fetus shared her abdominal cavity with the larger one. The digestive tract was shared up to the duodenum. Beyond duodenum both fetuses had separate tracts down to rectum with independent patent anal openings. The cecum, colon and rectum were also developed in both fetuses. The portion of intestine from jejunum to rectum was more developed in the smaller as compared with the larger fetus. Only two kidneys were present in the abdominal cavity. The right kidney was normal in position whereas the left kidney had moved anteriorly and was located at the point of junction of cranial extremity of the smaller fetus at the level of the diaphragm of the larger fetus. Slightly above the left kidney a small under- developed independent liver was found whereas a well-developed liver was present at the caudal aspect of the diaphragm to the right of the median plane where it is normally found. The spleen and pancreas were also present at normal positions.

**Fig. 2:**
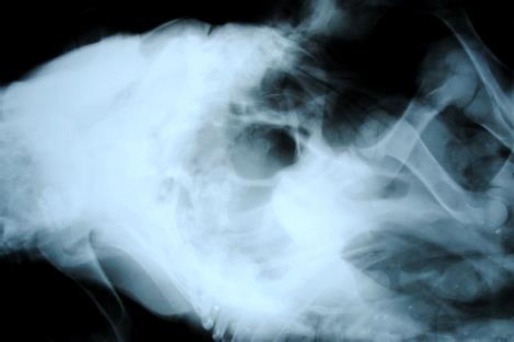
Radiographic Projections showing forelimbs, thoraco-lumbar spine and hind limbs of larger fetus.

**Fig. 3:**
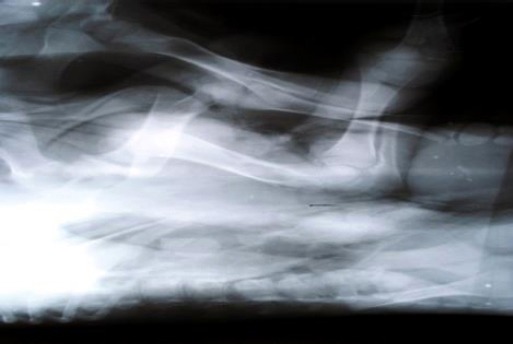
Radiographic Projections showing forelimbs of smaller fetus.

Radiographically, two different skeletons were visible in the monster in its thoraco-lumbar lateral and oblique ventro-dorsal radiographic projections. The primary larger fetus had properly developed forelimbs, a thoraco-lumbar spine as well as hind limbs (Fig. 2). The smaller fetus showed fully developed forelimbs composed of a scapula, humerus, radius, ulna and distal bones (Fig. 3).

The thoraco-lumbar spine was evident but was not very clear as all the vertebrae were apparently smaller, compressed and distorted in shape. However, eight ribs were identifiable which converged at its spine which was found to be attached at a relatively ventral aspect of the thorax of the larger fetus.

There were comparably fully developed pelvic bones found attached with the spine almost at the area from where the forelimbs originated. However, the correctness of the configuration of the pelvic girdle in the smaller fetus could not be ascertained due to the lack of a sufficient number of radiographic images.

## Discussion

Conjoined twins could be caused by a number of factors, which are influenced by genetic and environmental conditions. The embryonic disk starts to differentiate on the 13^th^ day of conception. If the split occurs after day 13, then the twins will share body parts in addition to sharing their chorion and amnion (Finberg, 1994).

The cause of anomalous development may occasionally be obvious but more often is obscure because of its multifactorial nature (Rousseaux and Ribble, 1988). Simon *et al.*, (2009) stated that conjoined twins were always genetically identical and shared the same sex. The present case seemed to be a non-inherited teratogenic defect of development with early complete duplication of cranial and caudal parts.
